# Functional sufficiency in VR: achieving non-corporeal embodiment

**DOI:** 10.3389/fnsys.2026.1778604

**Published:** 2026-02-27

**Authors:** Malcolm Wright, Olivia Petit, Alexander Schnack

**Affiliations:** 1 Massey Business School, Massey University, Auckland, New Zealand; 2 Ehrenberg Bass Institute for Marketing Science, Adelaide University, Adelaide, SA, Australia; 3 Kedge Business School, Marseille, France

**Keywords:** ecological validity, embodiment, functional sufficiency, immersive virtual reality, mental simulation, multisensory integration, perception-action loop, telepresence

## Abstract

This article provides a novel functionalist account of embodiment in immersive virtual environments, grounded in a formal model of cognition, supported by past empirical evidence, and offering a testable framework for predicting when virtual experiences will produce cognitive and emotional effects. Our approach complements existing work on telepresence and subjective experience by applying the Thin Model as an intermediate theory linking interface affordances to perception, emotion, and behavior. Drawing on previously published immersive virtual reality studies, we show that when key functional elements - such as sensing, recognition, inspection, and feedback - are preserved, behavioral and emotional outcomes remain stable even when locomotion mechanisms differ. These findings support a criterion of functional sufficiency for embodiment where interface substitution leaves core policies of action unchanged. We outline a set of theory-driven tests to identify the limits of this invariance and argue that embodiment should be defined by the integrity of the perception–action loop, not by anatomical mimicry.

## 1 Introduction

Although virtual environments may be seen by some as mere simulations, they provide domains in which real perception and action may still occur. Chalmers (2022) argues that virtual experiences and objects possess the same ontological and epistemic status as their physical counterparts: what matters is causal engagement, not the substrate generating perceptions. Petit et al. (2022) extend this perspective, emphasizing that conscious experience depends on the brain’s predictive modeling of incoming sensory inputs. Even when inputs are virtual, people can be expected to generate anticipatory models that allow them to perceive, feel, and evaluate objects continuously with real-world experience (Tal et al., 2026). Mental simulation, grounded in prior sensory and motor encounters, enables users to “fill in the blanks” imagining how an object might look, feel, or sound, while visual technologies can further enhance the vividness and personal relevance of these predictions (Petit et al., 2019). In this sense, virtual environments instantiate the functional processes of perception, evaluation, and reflection, expanding what it means to consciously experience.

Together, these views license treating virtual encounters as real enough to matter - not as illusions to be corrected, but as legitimate contexts in which consciousness, decision, and ethics unfold. This position aligns with Petit’s work on consumer digital consciousness, which explores how embodied perception and valuation operate across physical and virtual boundaries (Petit and Velasco, 2026). It also raises new questions for the conceptual analysis of embodiment. If the virtual is the real, how does it make sense to talk about embodiment?

Embodiment in VR encompasses three interrelated components: agency (the feeling of causing one’s actions), body ownership (the feeling that the virtual body is the source of sensations), and self-location (the spatial experience of being inside a body) (Guy et al., 2023). Embodiment is often tested by asking whether participants can move as they do in the physical world; whether they can walk, bend, or turn naturally. Yet this emphasis on physical mimicry mistakes motor replication for the cognitive activity and decision policies that guide behavior in the world outside the headset, despite evidence that the sense of embodiment is unaffected by alternative physical implementations (Dewez et al., 2020).

Our central claim is that embodiment depends on the preservation of a functional loop, rather than on anatomical fidelity, defined as the degree to which a virtual interface replicates the biomechanical and sensorimotor properties of the physical body. When a virtual interface preserves the structure of perception, recognition, and feedback, we argue people will act as they normally would, regardless of how their locomotion or other individual affordances are implemented. This would enable functional embodiment that allows VR environments to be more realistic simulacra of, or in some circumstances replacements for, regular interactions in the physical world. Embodiment, therefore, should be assessed at the level of policy - understood here in the reinforcement-learning sense: a learned mapping from perceived states to action selection - rather than at the level of mechanical resemblance. We develop this claim by drawing together formal theory, empirical evidence from immersive VR studies, and a set of testable propositions designed to identify where this sufficiency breaks down.

## 2 The Thin Model as functionalist intermediate theory

To develop our functionalist account of embodiment we draw on the Thin Model, an intermediate theory of the mind that provides a formal account of mental function grounded in four core axioms: materialism, functionalism, reductionism, and recursion (Wright et al., 2023). It defines an autonomous cognitive system through eight elements: a viable form, guiding tendencies or goals, sensory inputs, a concept store, recognition processes, action tendencies, feedback loops, and, optionally, a sense of self. This structure is designed to be substrate- and species-neutral, enabling analysis of cognition without requiring a human biological frame. As such, it provides a functional scaffold for understanding how technologically mediated environments can support perception, emotion, and decision-making, even when the underlying mechanisms differ from those found in the physical world.

The Thin Model provides a basis for identifying which cognitive functions must be supported for embodiment to occur. In immersive virtual environments, we can map its core functional elements directly onto the interface: sensory inputs correspond to rendered sights, sounds and/or haptic feedbacks; recognition requires stable and discriminative object identity and label clarity; action tendencies emerge through affordances for reaching, grasping, rotating, and inspecting; and feedback is provided through immediate outcomes and cumulative reinforcement. These functions enable the control loop at the heart of situated behavior: perceive → recognize → inspect → decide → update. When this perception-action loop is intact, users can enact stable patterns of attention and action regardless of how their movement through space or other affordances are implemented.

Taken together, these mappings suggest embodiment depends on sufficient functionality across the major elements of an autonomous entity, not on full–body mimicry. When the perceptual and behavioral elements of the loop are supported, users can sustain coherent policies of attention, evaluation, and action even as for example the physical means of locomotion change. The prediction follows directly: outcomes will remain invariant under interface substitution so long as the functional circuit linking perception, recognition, inspection, decision, and feedback remain intact. Where that circuit is disrupted - by degrading inspection, recognition, or feedback - embodied experience, affect, and behavior should diverge in measurable ways.

The Thin Model can be aligned with predictive processing and active inference frameworks, which have become influential in understanding perception, action, and embodiment in the brain (Tal et al., 2026). Predictive processing posits that the brain continuously generates hierarchical predictions about sensory inputs and minimizes prediction errors through perception and action (Clark, 2013; Friston et al., 2017). Embodied interactions in VR can therefore be understood as the brain’s engagement in minimizing discrepancies between expected and actual sensory states, even when those states are generated by artificial stimuli. Within this view, functional embodiment depends not on physical mimicry but on the preservation of sensorimotor contingencies that enable effective error minimization and confidence in action outcomes. This approach is present within the Thin Model in which sensory inputs are recognized through comparison with a concept store, generating feedback and action tendencies; however, the Thin Model is more general and accommodates a broader range of mechanisms than Active Inference alone.

Neural evidence suggests that prediction error signaling engages regions such as the anterior cingulate cortex (ACC), which tracks the mismatch between expected and observed events and supports cognitive control and learning (prediction error signals in ACC, Alexander and Brown, 2019). Oscillatory dynamics (e.g., beta and theta bands) and functional connectivity changes within sensorimotor and parietal networks have also been linked to agency and action–outcome coupling, revealing how neural communication patterns shift when prediction comparisons succeed or fail (Buchholz et al., 2019). These neural signatures can reveal disruptions in the perception–action loop, for example, elevated prediction-error-related activity or atypical oscillatory patterns, even when overt behavior remains stable. Such markers provide a testable basis for neural implementation of minimal embodiment in future fMRI studies, indicating whether the cognitive inference processes that sustain perception, agency, and decision-making are intact.

## 3 Minimal embodiment, defined

Having introduced the Thin Model and demonstrated its conceptual fit, we now propose a more precise definition of minimal embodiment. Our aim is to clarify what must be preserved at the interface level for virtual experience to support real behavior and emotion.

Minimal embodiment can be defined as the preservation of the task-relevant perception–action loop described in a general sense by the Thin Model - sensing, recognition, action tendencies, feedback, and optional self-monitoring, instantiated for situated behavior. When these functions are supported by the possession and exercise of sensorimotor skill (Noë, 2004) and appropriate affordances (Gross et al., 2005), an agent can perceive, evaluate, and act coherently within a virtual environment. Embodiment, on this account, is not a property of bodily duplication but of maintaining a closed functional circuit that allows adaptive behavior to unfold.

A closed functional circuit also contributes to other aspects of a meaningful virtual experience, such as minimal groundedness, the sense of connection to the environment, to others, and to temporal context. Minimal groundedness supports emotional stability, cognitive coherence, and engagement (Eichinger et al., 2022). When perception, inspection, feedback, and symbolic cues are preserved, users can feel anchored in familiar or socially meaningful contexts, allowing virtual interactions to engage both cognitive and affective dimensions (Xie and Desouza, 2025).

This framing shifts the focus of VR experiences from physical mechanics to functional and environmental sufficiency. Whether movement is enacted through walking, teleportation, or controller input is secondary to whether the interface affords stable perception, effective recognition, responsive feedback, and cues that connect users to meaningful places, people, and temporal anchors. If these affordances are intact, outcomes such as attention patterns, choice distributions, and affective engagement should remain within the same range that can be observed in physical contexts. Functional sufficiency, rather than sensorimotor completeness, becomes the relevant criterion.

## 4 Empirical anchors

**FIGURE 1:**
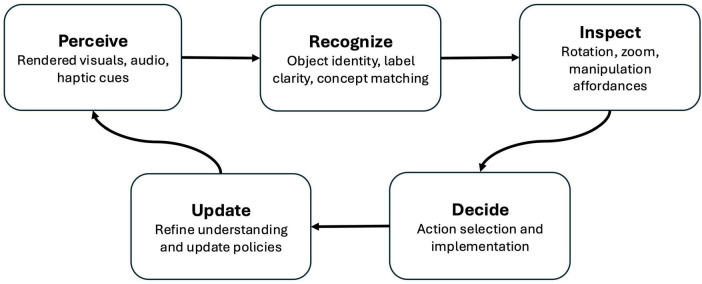
The perception-action loop in immersive virtual environments. The loop instantiates the Thin Model’s functional architecture for situated behavior in VR. Functional sufficiency is achieved when VR interface elements adequately support each stage, regardless of how specific affordances (e.g., locomotion) are implemented. Feedback from the update stage can modulate all other stages of processing.

Seen in this light, earlier empirical findings can be read as tests of function rather than of anatomy. The observed invariance of behavior and emotion across different locomotion modes in VR, for example, reflects preservation of the core perception-action loop (Figure 1). Variations in path or movement style are surface differences; what matters is that the cognitive system continues to operate within a coherent feedback structure. This realignment shifts the focus of embodiment research from reproducing form to identifying which functional elements sustain meaningful experience. We now briefly review illustrative literature, considering the impact of VR locomotion on embodiment, the use of immersive VR to enhance realism of shopper behavior, and the role of mental simulation in VR. The empirical examples presented here draw on consumer behavior research, reflecting the origins of our data. However, the functional framework applies wherever immersive systems support perception, recognition, and decision-making - including training, simulation, and entertainment contexts.

### 4.1 Embodiment

Several studies have investigated how changes in locomotion method impact perceived embodiment in virtual environments. Embodiment in VR is commonly understood as the extent to which users experience the virtual body as their own, encompassing feelings of body ownership, agency, and self-location. Locomotion techniques are often assumed to play a critical role in this process because they directly link bodily movement to spatial displacement and sensorimotor feedback. Empirical findings, however, suggest that the impact of locomotion on embodiment depends strongly on the perspective adopted in the virtual experience. For instance, Ulrichs et al. (2024) investigated three locomotion methods in a third person (3PP) virtual reality simulation and found that locomotion through natural movement (arm-swing) outperformed the two alternative methods, head-tilt and joystick. In contrast, studies testing changes to perceived embodiment in a first-person (1PP) virtual simulation using head-mounted displays have consistently shown that there was no difference in embodiment between locomotion methods. These studies tested joystick versus push-pull (Freiwald et al., 2022), real walking versus walking-in-place, and virtual steering (Dewez et al., 2020), and assisted cycling locomotion versus active cycling locomotion (Moullec et al., 2023). While more studies are needed to investigate other more popular locomotion methods, such as instant teleportation, these early findings consistently demonstrated that locomotion is not a primary driver of embodiment in first-person virtual experiences, whereas it plays a more salient role in third-person contexts where the user’s relationship to the avatar is externally mediated.

### 4.2 Behavior

Since studies have collectively failed to show that physical mimicry (locomotion) impacts embodiment, there are most likely no changes in user behavior to be expected. In early comparisons between desktop and immersive VR, consistent increases in presence proxies and perceived naturalness were observed when participants engaged with the environment through a head-mounted display and tracked controllers (Schnack et al., 2019). These findings suggest that immersive VR supports the necessary perceptual and interactive conditions to enable the full perception-action loop. When this loop is intact, participants behave in line with well-established shopper patterns (Schnack et al., 2020); purchase rates increase with shelf height, private-label products receive a plausible share of the basket, and unplanned purchases occur at realistic frequencies. These effects mirror physical-world observations and suggest that participants are not merely playing through a simulation but enacting familiar policies of attention, evaluation, and choice.

In a controlled comparison between motion-tracked walking and instant teleportation within immersive VR, participants followed different paths through the environment but produced statistically equivalent outcomes (Schnack et al., 2021a). Heatmaps revealed predictable differences in movement - such as central aisle bias among teleporting users - but these variations did not affect key behavioral or emotional measures. Basket size, trip duration, total spend, unplanned purchases, and uptake of unfamiliar brands all remained stable across locomotion types, and a subsequent study found no moderation by shopper personality (Schnack et al., 2021b). EEG traces of engagement, excitement, and stress also showed no significant differences. These findings suggest that when the functional loop is preserved - particularly recognition, inspection, and decision-feedback - changes to gross locomotor input do not alter underlying policies of action. Other researchers also found that locomotion had no impact on telepresence (Soler-Domínguez et al., 2020).

### 4.3 Mental simulation

The functional loop implied by the Thin Model has cognitive as well as behavioral elements (Wright et al., 2023). Some of these have been studied in VR, including mental simulation, the cognitive mechanism through which individuals internally recreate sensory and motor experiences associated with objects or situations, enabling better environmental understanding and facilitating decision-making (Barsalou, 2008, 2020). VR enhances mental simulation, particularly through first-person perspectives, which amplify its effects by aligning virtual perception with the user’s body schema. For instance, Basso et al. (2018) showed that first-person perspectives increase neural activity in regions associated with rewards (amygdala) and sensory-motor processing (superior parietal gyrus) during observation of hand actions, whereas third-person perspectives elicit weaker engagement. Immersive VR naturally leverages this mechanism: first-person visualization activates neural patterns associated with relevant sensory-motor experiences, allowing users to simulate outcomes and evaluate interactions even without direct physical input (Petit et al., 2019). These simulations are not mere imagination, they functionally replicate perception–action loops active during real-world interaction, guiding subsequent behavior and emotion.

Recent work highlights how Sensory-Enabling Technologies (SETs) can further amplify mental simulation in digital environments. By integrating multisensory cues, such as haptic feedback, temperature, wind, or olfactory signals, VR systems can more fully activate the sensory-motor patterns associated with physical interactions (Petit et al., 2019). For example, adding touch-enabled controls in VR increases engagement through haptic mental imagery, consequently influencing product evaluations (Cowan et al., 2021), while scent cues strengthen anticipatory emotions and states of flow, strengthening brand perceptions (Flavián et al., 2021; Cowan et al., 2023).

The fidelity of these simulations depends on multisensory congruence. Just as synchronous visual and tactile stimulation induces self-attribution in the rubber hand illusion (Botvinick and Cohen, 1998; Ehrsson et al., 2004), VR environments can manipulate body ownership, self-location, and interoception through synchronized cues, producing body transfer illusions where users perceive avatars as extensions of their own bodies. Self-relevant avatars are particularly effective in strengthening these functional loops (Petit and Velasco, 2026). Seo et al. (2017) found that participants exposed to avatars bearing their own faces experienced higher self-presence, both subjectively (self-report) and physiologically (EEG). Users paid more attention to self-relevant faces, reported greater identification, and exhibited neural markers of enhanced cognitive engagement. This illustrates that functional relevance to the user, not anatomical realism, is again the critical factor in sustaining the perception–action loop.

## 5 Implications for design and validity

This functional framing reshapes design priorities. If locomotion realism is not essential, but inspection fidelity is, then resources should flow accordingly. High-resolution labels, stable object identity, intuitive hand presence, and responsive object manipulation contribute directly to the perception–action loop (see, for instance; Jang et al., 2024; Luangrath et al., 2022; Xionghui et al., 2024). Avatars might be optimized to strengthen embodiment through behavioral expressiveness, or self-relevance, enhancing attention, engagement, and functional interaction, although avatar realism alone is insufficient to affect perceptions of virtual body ownership (Mal et al., 2024).

Designers can further leverage SETs to reinforce these cognitive loops. Multisensory enhancements, such as haptic gestures, tactile surfaces, olfactory cues, or wind effects, can enrich inspection and interaction, making virtual products feel more tangible and improving anticipatory evaluation (Amini Gougeh and Falk, 2023; Chen and Tsai, 2025). These interventions are particularly effective when visual cues alone are insufficient to support accurate mental simulation or when virtual experiences must align with real-world expectations (de Vries et al., 2018; Cowan et al., 2023).

The invariance of behavioral and emotional outcomes across different locomotion methods suggests that full-body motion fidelity is not always required for valid immersive experiences. Where the perception–action loop is preserved, teleportation can serve as a practical and effective means of navigation. Locomotion platforms may improve realism or comfort for specific users or tasks, but they are not necessary to support the functional criteria for embodiment (Dewez et al., 2020). Simulating locomotion by walking with full-body tracking offers diminishing returns when these proximal affordances are already in place.

The functional stability observed in immersive VR supports its use in behavioral research, market simulations, and applied training contexts. When the system preserves the relevant control loop, users enact decision policies consistent with physical-world patterns. This strengthens the case for immersive VR as a valid platform for studying situated cognition, provided the limits of sufficiency are clearly understood and empirically tested.

## 6 Boundary conditions (functional breakdown)

As environments grow in scale or complexity, locomotion constraints can begin to shape what participants see and attend to Gabay and Schonberg (2025). In large virtual stores or settings with high product novelty, teleportation may reduce shelf exposure and truncate exploration paths. This can shift decision outcomes by limiting the information available for recognition and evaluation. In such cases, the perception–action loop may remain partially intact but insufficiently exercised, leading to measurable deviations in unplanned purchases or brand switching. Functional sufficiency, here, becomes a question of reach; not in a motor sense, but in terms of what the system enables users to encounter. One solution – or comparative test condition - could be to fix targets on the map and allow the user to only teleport to adjacent targets (Freiwald et al., 2022).

Embodied decision-making also depends on effective object recognition, which is impacted by factors such as the level of control over a virtual object (Wang and Datta, 2010). This control affords the user’s ability to closely inspect an object and retrieve object-specific information relevant for decision-making. However, when mechanical inspection features such as rotation and zoom features are degraded, the object recognition component of the loop is impaired by reducing interactivity. Several studies have shown that this degradation in interactivity in turn negatively impacts user behavior (Kang et al., 2020; Kim et al., 2021).

The same issue can occur when the visual representation of virtual products is suboptimal. For instance, situations when label clarity is degraded can lead to a reduction in vividness and readability, which in turn reduces a user’s affordance of object recognition. While Kang et al. (2020) found no effect of graphics quality on informativeness or decision-making, other researchers did find an existing relationship between graphical quality, perceived vividness, spatial presentation (2D vs. 3D) and decision-making/user behavior (Kim et al., 2021).

Object recognition further depends on the context of the environmental cues (Oliva and Torralba, 2007), requiring thoughtful planning around the presence of surrounding objects. In immersive store contexts, impaired object recognition is likely to affect the uptake of unfamiliar brands, reduce user confidence, and lower presence ratings (Kim et al., 2021). These effects are not artifacts of interface quality *per se*, but direct consequences of interrupting the cognitive processes that support value inference and choice. Inspection is a bottleneck for cognition; when lost, the system no longer supports the responses it is meant to elicit.

Importantly, the type of product category shapes which inspection cues are most relevant (Bigne et al., 2024). For functional objects, such as packaged products, precise manipulation and detailed views, such as zooming or rotating, are critical to simulate assessment of suitability and perceived risk. In contrast, for experiential products like served foods, the broader context of the environment and first-person visualization play a more significant role than detailed object interaction. In these cases, providing a familiar or contextually rich VR environment may enhance mental simulation more effectively than improving mechanical interaction alone. Immersive retail design should therefore calibrate product and environmental cues to product characteristics, ensuring that inspection and interaction support the specific product type requirements (Petit et al., 2022).

The framing of the task also matters. Participants given a goal-directed assignment may experience heightened stress or urgency compared to those in exploratory or recreational conditions, even when the environment is the same. In previous studies, engagement and excitement showed consistent early peaks (Tian et al., 2022), but stress trajectories varied with the framing of the interaction (Ban et al., 2024). This suggests that the feedback element of the loop is modulated not only by interface mechanics but by perceived intention and consequence. Here, embodiment can be expected to shift with task valence - not because the loop breaks, but because it recalibrates.

## 7 Research program: theory-driven probes

To test the functionalist account of embodiment, we propose a focused set of experimental manipulations, each targeting a distinct component of the perception–action loop. The aim is not to evaluate immersive technology in general, but to probe which interface elements are necessary for stable policies of attention, emotion, and behavior. Each test examines the boundary between functional sufficiency and breakdown, guided by the Thin Model’s mapping of sensing, recognition, inspection, and feedback.

**Test 1: Degrade Inspection**

Reduce the clarity of product labels and disable object rotation. This targets recognition and inspection, and should directly affect uptake of unfamiliar brands, increase decision latency, and lower presence scores. If participants cannot resolve the relevant distinctions, policy formation should degrade.

**Test 2: Expand Scale**

Increase store size and assortment novelty. This tests whether locomotion mechanisms that limit spatial exposure (e.g., teleportation) compromise the availability of perceptual input. If exposure falls below a functional threshold, outcomes such as unplanned purchases and diversity of choice should diverge from behavioral benchmarks.

**Test 3: Manipulate Multisensory-inputs**

Compare conditions with minimal sensory input (visual-only) versus enriched multisensory environments using SETs. This can include ambient audio, haptic feedback, temperature changes, wind, and scent cues, while keeping visuals constant. The goal is to test whether enhanced sensory congruence strengthens mental simulation, presence, and engagement (e.g., anticipatory imagery of product use or consumption outcomes). We predict richer multisensory input will increase presence and immersive experience, supporting the perception–action loop, but may not change core behavioral outcomes unless the additional cues influence perceived salience or product relevance. This manipulation also allows exploration of which sensory modalities are most critical for sustaining minimal embodiment and effective decision-making.

**Test 4: Alter Hand Fidelity**

Remove or degrade the ability to grasp, rotate, or reposition items. This affects the physical realization of action tendencies while leaving recognition cues visible. If object handling is critical to embodied evaluation, we expect a drop in product engagement and a shift in choice patterns.

**Test 5: Grounding Cues Manipulation**

Introduce or remove contextual cues that connect users to familiar places, social actors, or historical references. Measure effects on emotional engagement, perceived stability, and decision confidence. This allows examination of whether groundedness emerges from minimal embodiment, supporting functional engagement.

**Test 6: Avatar Customization**

Manipulate the degree of avatar personalization and resemblance to the user, from generic avatars to highly self-representative avatars. We predict self-resembling avatars will strengthen embodiment by enhancing perception–action loops, increasing emotional engagement, and reinforcing stable behavioral policies. This test examines whether the alignment of virtual self-representation with the user is a functional component of minimal embodiment.

Together, these tests allow for potential falsification of the minimal embodiment hypothesis. Where outcomes shift despite preserved recognition and inspection, the hypothesis is undermined. Where outcomes remain stable despite surface-level interface changes, it gains support.

## 8 A compact measurement stack

In support of these tests, we suggest a specific measurement stack that explicitly distinguishes embodiment from telepresence. This distinction is grounded in the theoretical understanding of the perception–action loop: before meaningful assessments of behavioral stability or functional embodiment can be made, the interface must support coherent sensing, recognition, and feedback. In other words, the loop must be operable for embodiment to manifest, making presence a necessary enabling condition rather than a direct indicator of embodied outcomes (Slater, 2009; Kilteni et al., 2012).

Established instruments such as the Igroup Presence Questionnaire (IPQ; Schubert et al., 2001) or the Presence Questionnaire (Witmer and Singer, 1998) can be used alongside usability measures (e.g., SUS; Brooke, 1996) to assess ease of movement, naturalness of interaction, and perceived immersion. These measures do not index embodiment *per se* but instead ensure that the participant’s perceptual and motor environment is coherent enough for the perception–action loop to operate. Only once these prerequisites are met can we interpret subsequent behavioral, physiological, and affective outcomes as reflecting the integrity of embodiment.

Embodiment itself is measured through convergent methods targeting ownership, agency, and self-location. Standardized questionnaires (VEQ; Roth and Latoschik, 2020) capture subjective experience, while behavioral tasks (response to threat, locomotion, mental imagery; Bourdin et al., 2017; Lenggenhager et al., 2007) provide objective evidence. Implicit agency measures such as intentional binding and sensory attenuation (Gentsch and Schütz-Bosbach, 2011) further validate control over actions.

Physiological measures provide complementary insight into the affective state of participants. EEG indicators of engagement, excitement, and stress offer a coarse but useful trace of affective state (Kober et al., 2012). Similarly, heart rate variability (HRV) and skin conductance (electrodermal activity) can provide additional coarse-grained evidence of arousal and stress responses, while eye-tracking data, including fixation duration and saccade patterns, can also offer insight into attention allocation and cognitive load (Halbig and Latoschik, 2021). They are best interpreted as secondary evidence that complements behavioral and self-report measures. In our locomotion comparison, the stability of these EEG measures across conditions reinforces the claim that emotional engagement depends on the integrity of the functional loop rather than on the specific form of locomotion.

Spatial metrics such as heatmaps, dwell time, and route entropy capture how participants traverse the environment. These indicators reveal surface differences in navigation while allowing assessment of whether such differences influence exposure or decision outcomes. For example, differences in path length or central-aisle bias may appear in teleportation versus walking conditions, yet if purchase patterns, interaction frequency, and affective states remain constant, this indicates that the underlying behavioral policy, and by extension the embodied function has been preserved. Additional measures such as object interaction counts, inspection times, or virtual gaze-based engagement including pupil dilation can provide finer-grained confirmation of functional loop integrity.

## 9 Conclusion

Embodiment in virtual environments should be defined by function, not by form. When systems support perception, recognition, inspection, and feedback, they enable behavioral and emotional engagement even when interface mechanics differ from those of the physical world. The Thin Model provides a principled way to identify which functions must be preserved and where substitution may lead to breakdown. This allows us to treat virtual experience not as a lesser analogue but as a cognitively valid domain. If policies remain stable, the experience is real enough to matter, psychologically, practically, and ethically. In designing immersive systems, we should follow the structure of the mind, not the body.

The functional sufficiency criterion extends beyond immersive VR. First-person videogames, desktop simulations, and other mediated environments may support embodiment to the degree that they preserve the perception-action loop. The relevant question is not the level of immersion but whether sensing, recognition, inspection, and feedback remain intact. This generalization invites comparative research across interface types.

Mental simulation is a central function that minimal embodiment must support. It allows users to anticipate outcomes, evaluate interactions, and enact real-world-like policies by internally reenacting sensory and motor experiences. Minimal embodiment should facilitate this process. VR, particularly through first-person perspectives and enriched environmental representations, is well suited to support mental simulation, especially when sensory inputs are coherently integrated, strengthening the perception–action loop.

Finally, if immersive systems reliably influence attention, emotion, and decision-making, they demand ethical consideration regardless of their ontological status. The fact that virtual environments are mediated does not exempt them from obligations around informed consent, data minimization, and debriefing. A functionalist account of embodiment reinforces this view: if the system supports meaningful cognition, it must be treated as a site of real psychological engagement.

## Data Availability

The original contributions presented in the study are included in the article/supplementary material, further inquiries can be directed to the corresponding author.
